# Signal Enhancement of Pressure-Sensitive Film Based on Localized Surface Plasmon Resonance

**DOI:** 10.3390/s21227627

**Published:** 2021-11-17

**Authors:** Bei Zhou, Feng Gu, Yingzheng Liu, Di Peng

**Affiliations:** 1 Key Lab of Education Ministry for Power Machinery and Engineering, School of Mechanical Engineering, Shanghai Jiao Tong University, 800 Dongchuan Road, Shanghai 200240, China; sabine1701@sjtu.edu.cn (B.Z.); 707996506@sjtu.edu.cn (F.G.); yzliu@sjtu.edu.cn (Y.L.); 2 Gas Turbine Research Institute, Shanghai Jiao Tong University, 800 Dongchuan Road, Shanghai 200240, China

**Keywords:** pressure-sensitive paint, silver nanoparticles, localized surface plasmon resonance, signal enhancement

## Abstract

Pressure-sensitive films have been used for measurement in micro flow, but thin films have very limited intensity, resulting in poor signal-noise ratio (SNR). This paper presents a pressure-sensitive film whose emission signal is enhanced by silver nanoparticles (AgNPs) based on localized surface plasmon resonance (LSPR). Electronic beam evaporator and annealing furnace are used to fabricate silver nanotexture surface. PtTFPP and polystyrene are dissolved in toluene and then spin-coated on the silver nanotexture surface to prepare the pressure-sensitive films. Signal enhancement of film with AgNPs due to LSPR is analyzed and enhancement effect of samples with different particle sizes and spacer thickness are compared. Pressure and temperature calibrations are performed to assess the sensing performance of pressure-sensitive films. Pressure-sensitive films with AgNPs demonstrate signal enhancement due to LSPR and show promise for measurement in micro flow.

## 1. Introduction

Pressure-sensitive paint (PSP) is a non-intrusive optical pressure measurement method based on oxygen-quenching mechanism. It has been widely applied in the aerodynamic testing [1,2,3,4]. PSP is usually composed of luminescent dyes as probe and polymer as binder, which is coated on the surface. Due to the small size of the molecules as functional probe, it is possible to measure oxygen concentration or pressure in micro flow [5]. It shows clear advantages over the conventional method such as pressure taps, which are difficult to apply in micro flow and provide insufficient information on the flow features. PSP has been applied in micro-nozzle measurement [5,6], but conventional PSP has a sub-millimeter spatial resolution, which is insufficient in the measurement of a micro flow. Matsuda et al. introduced a pressure-sensitive molecular film fabricated with Langmuir-Blodgett technique and applied the film in micro-nozzle measurement [7,8,9,10]. For PSP measurement in micro flow, a main challenge is that the pressure-sensitive film has limited luminescent intensity because the film is very thin (often of the scale of micrometers or nanometers). Improvements are required to enhance the PSP intensity for better signal-noise ratio (SNR) in measurement.

Localized surface plasmon resonance (LSPR) is a phenomenon that surface conduction electrons of nanoscale metal particles oscillate when excited by electromagnetic radiation. Willets et al. reviewed comprehensively on the theory and application of LSPR and discussed the fabrication of metallic nanoparticles and spectroscopy measurement [11]. Incident light interacts with particles much smaller than the wavelength of incident light, and surface plasmon of the particle oscillates at a LSPR frequency [12], resulting in an electromagnetic field that further enhances the scattered signal. Luminescent molecules near the metallic nanoparticle are in such dielectric environment that their radiative emission is enhanced, coupled with the resonance of the metal particle. This phenomenon is also referred to as surface enhanced fluorescence (SEF) or metal enhanced fluorescence (MEF). Early study on the interaction between solid surfaces and molecular fluorescence was conducted by Chance et al. [13]. Gersten and Nitzan gave a mathematic model for the dynamics of energy transfer when molecules are near a particle and concluded that the process is sensitive to molecular parameters [14]. Anger et al. provided experiment results on the enhancement and quenching of single molecule fluorescence in vicinity of a gold nanoparticle [15]. The results showed that the emission was enhanced when the distance of the molecule and particle was larger than 5 nm. However, the emission was quenched when the molecule was too close to the particle.

Several previous studies have shown the possibility of combining metallic nanoparticles with PSP for signal enhancement. Pan et al. observed a 200-fold enhancement of PtOEP phosphorescent emission on a nanotexture silver surface [16]. Miura et al. introduced LSPR into PSP fabrication and reported that by adding 35 nm silver particles in PSP, luminescent emission was enhanced by about three times [17]. Peak and Watkins added silver nanoparticles coated with a silica shell into PSP and reported an enhancement factor of more than 1.5 [18]. Problems remain, in that the signal enhancement is limited in conventional PSP. In addition, the fabrication of PSP with controlled distance between nanoparticles and luminescent molecules is difficult.

In this paper, a new type of pressure-sensitivity film with silver nanoparticles (AgNPs) is proposed to improve SNR in micro flow measurement. The distance between nanoparticles and luminophores is accurately controlled by a spin-coated polymer layer. Signal enhancement of the film due to LSPR is evaluated by comparing pressure-sensitive films fabricated with and without AgNPs. The mechanism of signal enhancement of pressure-sensitive films by LSPR and the relation between emission intensity enhancement and lifetime are studied and discussed.

## 2. Materials and Methods

In order to obtain surfaces with silver nanostructure, physical and chemical approaches are available [19]. Chemical synthesis of AgNPs usually involves oxidation-reduction reaction, while physical methods consume electrical and thermal energy. The physical approach is preferred over the chemical reduction method in this paper due to better particle size uniformity and higher purity [19]. Ag was deposited with a rate of 0.5 Å/s by an electronic beam evaporator (Kurt J. Lesker PVD75) on a silicon wafer. Then, Ag-deposited silicon wafers were put in a tube furnace and went through a thermal annealing process in a nitrogen-protected environment. Different annealing temperatures result in different morphology of silver on the surface, which will be further illustrated in the result section.

**Figure 1:**
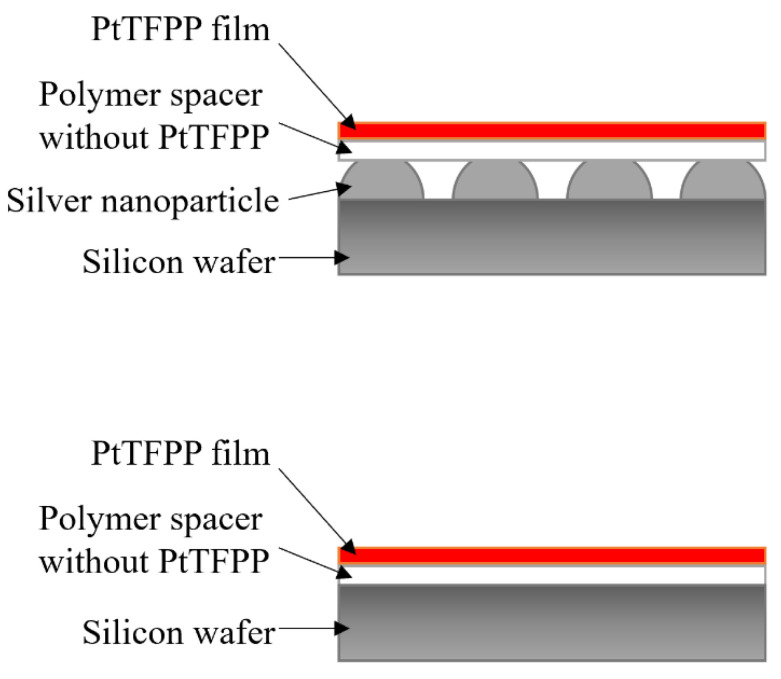
Microstructures of pressure-sensitive films with (**top**) and without (**bottom**) AgNPs.

**Figure 2:**
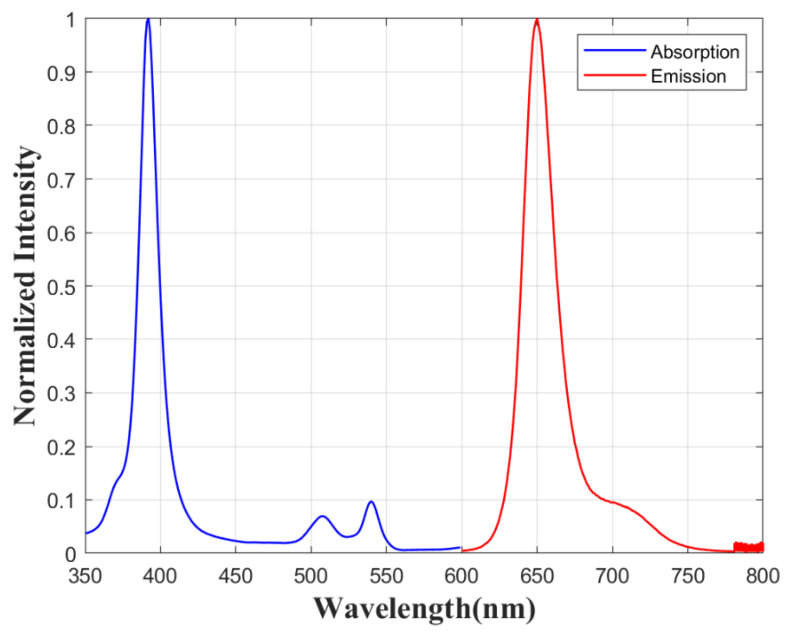
Absorption and emission spectra of PtTFPP [21].

To assess the signal enhancement by silver nanoparticles (AgNPs) in vicinity to luminophore, pressure-sensitive films with AgNPs and reference samples without AgNPs were prepared. Structural scheme of the film is shown in Figure 1. A transparent spacer is required between AgNPs and the luminophore to keep the distance and prevent luminescence quenching. Platinum(II)-5,10,15,20-tetrakis-(2,3,4,5,6-pentafluorphenyl)-porphyrin (PtTFPP) is selected as luminescent molecule of pressure-sensitive film. PtTFPP is a common luminophore used in PSP due to its high pressure sensitivity and good photostability [1,2,20]. The absorption and emission spectra of PtTFPP are plotted in Figure 2 [21].

To prepare the spacer, polystyrene was first dissolved in toluene. Then, the solution was spin-coated on the silicon wafers with and without AgNPs. Spacers with different thickness were fabricated to study the effect of distance in emission enhancement. Thickness of the spacer was measured on a clear silicon plate with atomic force microscope (AFM, MFP-3D, Oxford Instruments, Oxford, UK). For every sample, the thickness was measured at three random locations, and the averaged results are shown in Table 1. With different polymer concentrations, film thickness can be controlled when spin-coated at a certain rotating speed (4000 rpm). Spacer layers were fabricated with thickness from 10 nm to 195 nm by varying the polymer concentration. For the PtTFPP film, 0.01 g/mL polystyrene and 1 mg/mL PtTFPP were dissolved in toluene. The solution was spin-coated at 4000 rpm above the spacer layer for all the samples. The thickness of PtTFPP film was about 30 nm.

Lifetime and intensity of the samples were measured to study the localized surface plasmon resonance effect of silver nanoparticles on the luminescence of PtTFPP. Lifetime of the films was measured with a photomultiplier tube (PMT, Hamamatsu h9305-03) and a 405 nm laser (OEM-HD-405-5W, Changchun New Industries Optoelectronics Tech. Co., Ltd., Changchun, China). For luminescence intensity measurement, samples were excited by a 385 nm UV-LED light source (UHP-T-LED-385, Prizmatix, Israel) and emission signals were collected by a scientific CCD camera (pco. 1600, pco. imaging) through a 50 mm lens (50 mm/f1.2, Nikon). A 650 ± 25 nm filter was mounted before PMT and camera lens to eliminate the excitation light signal. Lifetime measurement was repeated five times for each sample and intensity images were the average of 20 images.

Static calibration of pressure-sensitive films was also performed to examine the pressure and temperature sensitivity. Samples were put inside a calibration chamber where pressure and temperature of the environment can be controlled. Luminescence signals were captured by the same CCD camera as previously mentioned. The pressure calibration range was from 40 kPa to 160 kPa. The temperature calibration range was from 25 °C to 50 °C. The refence pressure and temperature were 100 kPa and 25 °C, respectively. Both pressure and temperature calibrations were repeated three times and errors were calculated. It should be noted that dynamic calibration was not performed at this stage, because the signal level of pressure-sensitive film was still insufficient for dynamic measurement.

## 3. Results and Discussion

### 3.1. Microscopy of Pressure-Sensitive Film Samples

**Figure 3:**
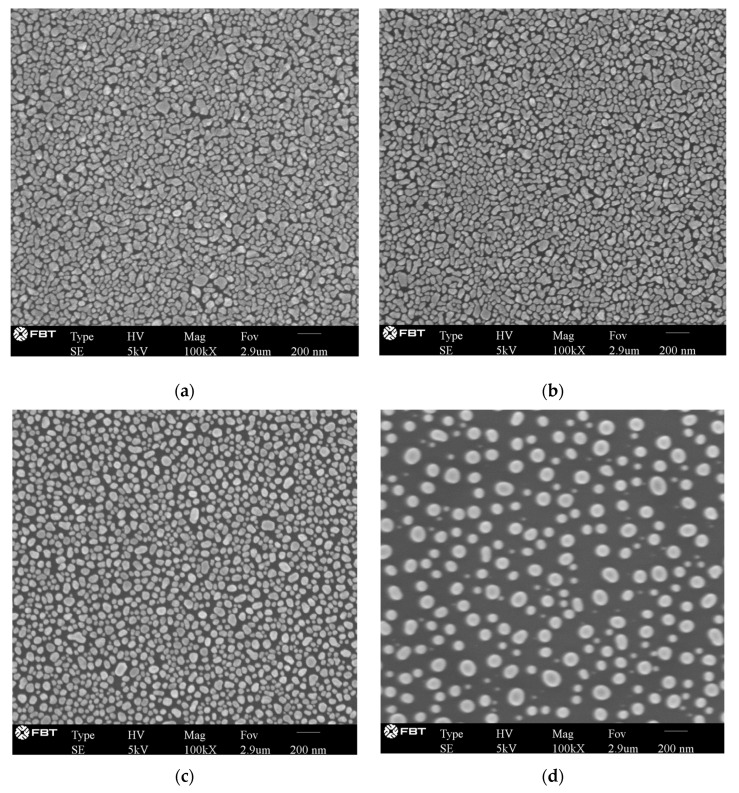
Morphologies of silver nanoparticles formed after difference thermal treatment process: (**a**) with no thermal treatment; (**b**) annealed at 80 °C; (**c**) annealed at 150 °C; and (**d**) annealed at 300 °C.

**Figure 4:**
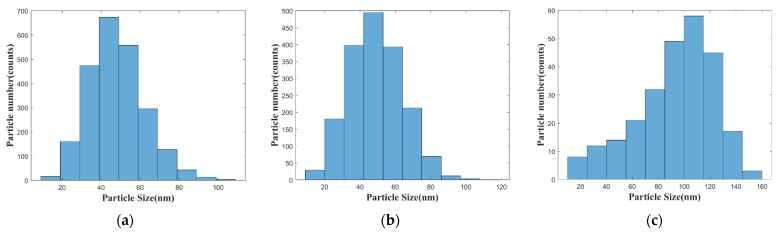
Histogram of AgNPs particle sizes after different thermal treatment process: (**a**) annealed at 80 °C; (**b**) annealed at 150 °C; and (**c**) annealed at 300 °C.

Morphologies of Ag-deposited silicon wafers after annealing in the furnace with different annealing temperature were observed with a scanning electron microscope (SEM, Navigator-100, Focuse Beam Technology, Beijing, China). As shown in Figure 3, the silver particles become bigger in size and more distant with each other with higher annealing temperature due to the agglomeration of particles. Histograms of particle sizes are plotted in Figure 4. Particles were approximately regarded as spheres. The diameters of sphere particles were calculated based on the area shown in the SEM images. For the samples annealed at 80 °C and 150 °C, sizes of most particles fall in the range of 40–50 nm, while for the sample annealed at 300 °C, sizes of most particles fall in the range of 100–120 nm. The effect of particle size on the localized surface plasmon resonance can be studied based on these AgNPs-coated substrates.

### 3.2. Lifetime and Intensity of Pressure-Sensitive Film Samples

**Figure 5:**
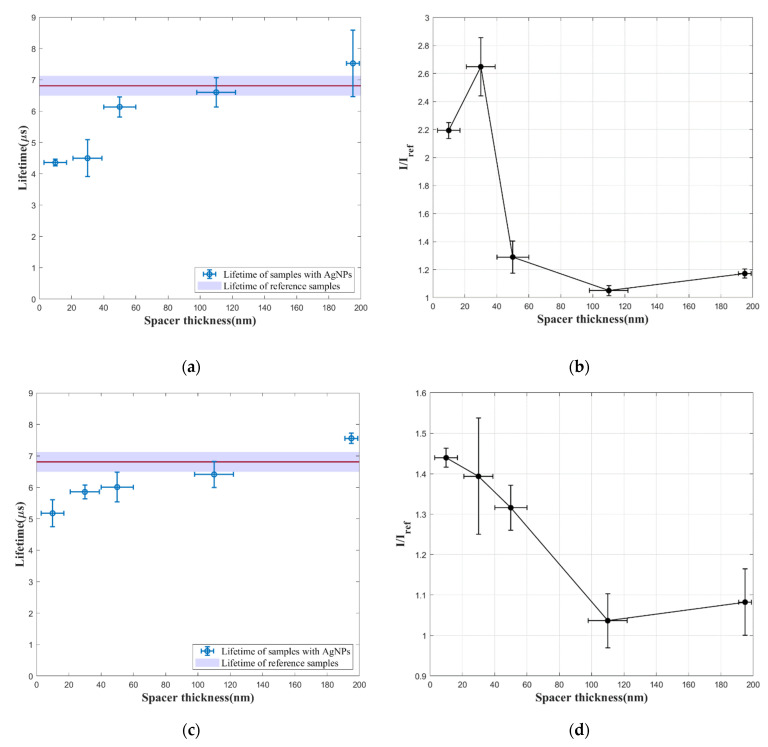

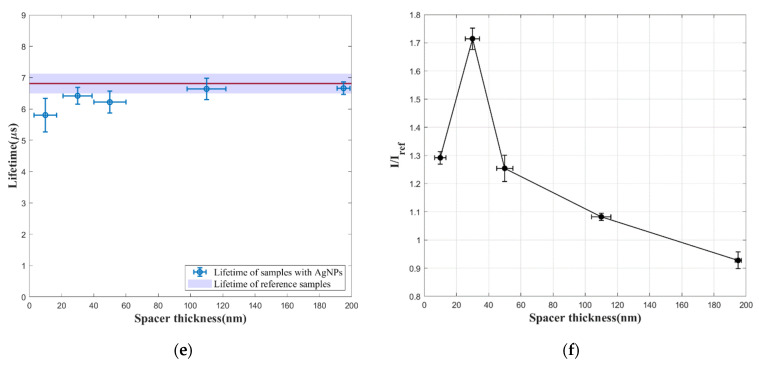
Lifetime and luminescence intensity of pressure-sensitive film samples with different spacer thickness and different thermal treatment process and are compared with reference samples without AgNPs: (**a**,**b**) samples of deposited Ag annealed at 80 °C; (**c**,**d**) samples of deposited Ag annealed at 150 °C; (**e**,**f**) samples of deposited Ag annealed at 300 °C.

Lifetime and intensity of the samples with AgNPs of different particle sizes and different spacer thickness are compared with the reference samples without AgNPs. The results are illustrated in Figure 5. The red line represents the average lifetime of five reference samples without AgNPs, and the purple area shows the error of the measurement. Each data point in the luminescence intensity plot shows the intensity ratio between the sample with AgNPs and the reference sample (with spacer of the same thickness). The uncertainty of film thickness is also shown for each sample, which was calculated from three measurements using AFM. The variations in the lifetime and intensity provide the basis to examine the signal enhancement effect of LSPR on the luminescence of PtTFPP.

According to the lifetime results in Figure 5a,c,e, films with AgNPs with 10 nm spacer for all particle sizes show an obvious decrease in luminescence lifetime compared to the reference sample (without AgNPs). The decrease in lifetime is less obvious when the spacer layer becomes thicker, until no significant difference is shown by samples with spacer of 195 nm thickness. It can also be observed that the smallest silver particles lead to the most decrease in lifetime with the small spacer thickness (<40 nm). The lifetime decrease indicates the occurrence of LSPR due to the silver particles. Gartia et al. provided fluorescence-lifetime imaging microscopy (FLIM) results of different dyes on nanoplasmonic substrate surface [22]. They pointed out that the additional pathway of de-excitation due to the proximity of the metal particle can explain the shortened lifetime. Although the presence of the silver particles enhances the electro-magnetic field, radiative and non-radiative decay rate of luminophore increases, leading to decreased lifetime.

Then, signal enhancement of samples with AgNPs is directly demonstrated by the intensity ratio results in Figure 5b,d,f. The intensity enhancement is obvious for small spacer thickness (<40 nm), and the maximum enhancement is 2.6-fold (AgNPs annealed at 80 °C, spacer thickness of 30 nm). The enhancement effect weakens as the distance between AgNPs and PtTFPP grows. There is some degradation of the signal enhancement at the smallest spacer distance (10 nm), which is likely due to the quenching of PtTFPP by the silver particles. This is in agreement with previous findings by Anger et al. [15]. For spacer layers of the same thickness, samples with AgNPs annealed at 80 °C (smallest particle size) are best enhanced, while the ones with larger particle size demonstrate less signal enhancement.

**Figure 6:**
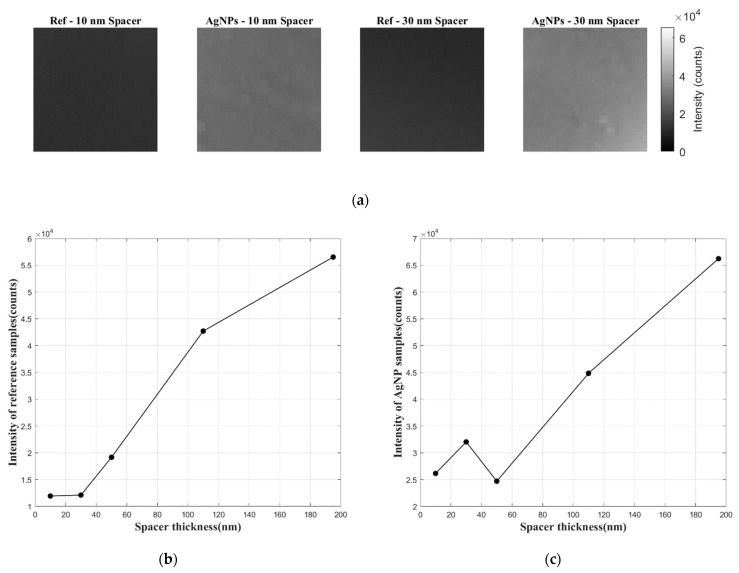
Comparison of the absolute intensity of pressure-sensitive films with different spacer thickness: (**a**) intensity images of samples with 10 nm and 30 nm spacer; (**b**) reference films with no AgNPs; (**c**) films with AgNPs.

In addition, it is important to note that although films with AgNPs show considerable signal enhancement compared to the reference samples with same spacer thickness, the emission signal of reference samples becomes stronger with thicker spacer layer (see Figure 6b). An explanation for this phenomenon is that when luminescent molecules are too close to the solid substrate surface, the luminescence is quenched. Thus, it is evident from Figure 6c that the absolute intensity of films with AgNPs is actually the largest when the spacer layer is 195 nm thick. This indicates that the pressure-sensitive film with a thick spacer and no signal enhancement may still be preferred during measurement, if the film thickness does not affect the flow.

### 3.3. Static Calibration of Pressure-Sensitive Film Samples

**Figure 7:**
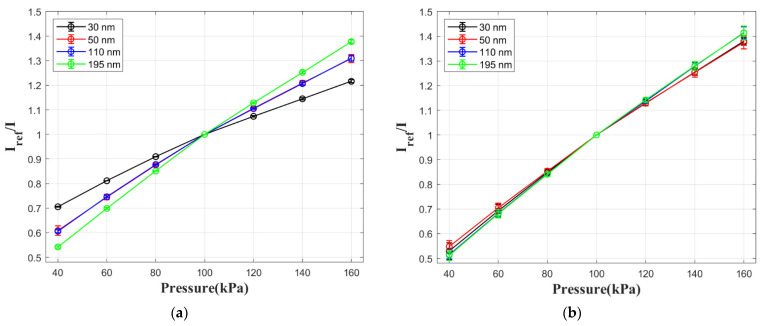
Pressure calibration results of pressure-sensitive films with different spacer thickness: (**a**) films with AgNPs; (**b**) reference films with no AgNPs.

Pressure calibration results are presented in Figure 7 for both pressure-sensitive films with AgNPs and reference films without AgNPs. The pressure sensitivity values in Table 2 are calculated by ∆(I_ref_/I)/∆P with the data at 40 kPa and 160 kPa. The samples with 10 nm spacer are not tested due to luminescence quenching as explained in the previous section. According to Figure 7b and Table 2, the spacer thickness has little influence on the pressure sensitivity of reference samples. However, the pressure sensitivity is reduced to 60% for films with AgNPs when the spacer is 30 nm thick. This is related to the increased non-radiative decay rate by an additional pathway of luminescent decay, when PtTFPP is in vicinity of AgNPs. A possible explanation for the change in pressure sensitivity is that the emission signal from this additional pathway is not quenched by oxygen. This leads to a lower pressure sensitivity for samples with AgNPs. When the spacer becomes thicker, the LSPR effect becomes weaker, so pressure sensitivity of films with AgNPs is in accordance with the reference sample with the same spacer thickness.

**Figure 8:**
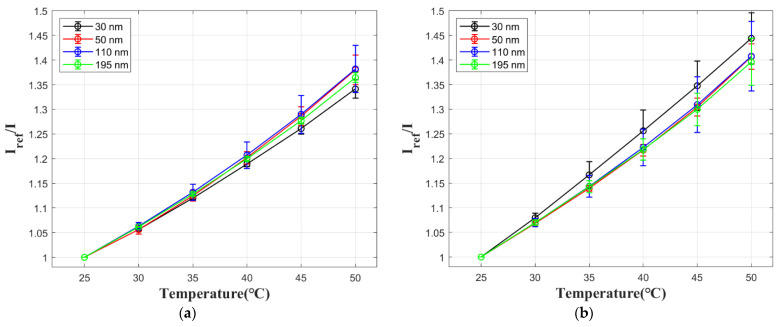
Temperature calibration results of pressure-sensitive films with different spacer thickness: (**a**) films with AgNPs; (**b**) reference films with no AgNPs.

Temperature calibration results are presented in Figure 8 for both films with AgNPs and reference samples without AgNPs. The temperature sensitivity values in Table 3 are calculated by −∆(I/I_ref_)/∆T with the data at 25 °C and 50 °C. Temperature sensitivity of the film shows no significant change for different spacer thickness. Pressure-sensitive films with AgNPs are slightly less affected by temperature compared with the ones with no AgNPs, but the values are quite close and do not show a particular trend. This indicates that temperature sensitivity of the film is not significantly affected by LSPR.

## 4. Conclusions

Pressure-sensitive films were fabricated on substrates with and without silver nanoparticles. A spacer (polymer layer) was used to adjust the distance between the pressure-sensitive film and the silver particles. Signal enhancement was observed for samples with spacer thickness of 10–120 nm. The largest enhancement (2.6-fold) was found when the particle size was 50 nm in average and the spacer thickness was 30 nm. LSPR became weaker when the distance increased, while luminescence was quenched when the pressure-sensitive film and silver particles were too close (10 nm). The enhanced intensity was accompanied with the decrease in luminescence lifetime. Pressure sensitivity declined as LSPR effect became stronger, while temperature sensitivity was not significantly affected. Future research should focus on applying the pressure-sensitive film for the measurement in micro flow.

## Figures and Tables

**Table 1:** Thickness of spin-coated films with different polymer concentration at a rate of 4000 rpm.

Polymer Concentration (g/mL)	Thickness of Spin-Coated Films (nm)
0.005	10 ± 7
0.010	30 ± 9
0.015	50 ± 10
0.025	110 ± 12
0.035	195 ± 4

**Table 2:** Pressure sensitivity of all the pressure-sensitive film samples.

Pressure Sensitivity (%/kPa)	30 nm Spacer	50 nm Spacer	110 nm Spacer	195 nm Spacer
Film with AgNPs	0.43	0.58	0.59	0.70
Film without AgNPs	0.71	0.69	0.75	0.75

**Table 3:** Temperature sensitivity of all the pressure-sensitive film samples.

Temperature Sensitivity (%/°C)	30 nm Spacer	50 nm Spacer	110 nm Spacer	195 nm Spacer
Film with AgNPs	−1.02	−1.02	−1.11	−1.07
Film without AgNPs	−1.23	−1.16	−1.16	−1.14

## Data Availability

The study did not report any data.
